# Editorial: Open Preperitoneal Mesh Repair for Inguinal Hernia – New Evidence, Old Arguments

**DOI:** 10.3389/jaws.2025.15294

**Published:** 2025-08-12

**Authors:** Frederik Berrevoet

**Affiliations:** Department of General and HPB Surgery and Liver Transplantation, Ghent University Hospital, Ghent, Belgium

**Keywords:** preperitoneal, inguinal hernia, open surgery, mesh, surgical technique

## Introduction

Inguinal hernia repair is one of the most common surgical procedures worldwide, yet the quest for further optimization of techniques remains an ongoing debate. Traditionally, the approach to inguinal hernias has relied heavily on open anterior mesh-based techniques or laparoscopic (posterior) mesh placement. However, the open preperitoneal approach has emerged as a compelling alternative, offering distinct advantages in terms of access, low recurrence rates, and excellent patient outcomes regarding chronic pain specifically. This special issue seeks to explore the benefits, challenges, and future directions of open preperitoneal techniques in inguinal hernia repair.

The open preperitoneal technique has already quite a history and in a narrative review by Soler this evolution in technique and approaches is clearly highlighted. Accessing the hernia through an incision in the lower abdomen, exposing the preperitoneal space, and placing a mesh in the space between the peritoneum and the abdominal wall, this technique offers a direct view of the hernia defect while avoiding many of the limitations associated with more conventional approaches.

Secondly, Lorenz et al. illustrated both the need for standardization of techniques as well as the potential advantages of the different open preperitoneal techniques versus the “old establishment” as the Lichtenstein repair and the laparoscopic techniques. Their conclusion is that open preperitoneal approaches for groin hernia repair are straightforward and safe, often yielding results comparable to, or better than other techniques.

Despite its promising benefits, the open preperitoneal technique is not without its challenges. The technique requires advanced knowledge of abdominal anatomy, particularly the preperitoneal space, which can be more difficult to navigate than the more familiar peritoneal or retroperitoneal spaces. This might be one of the main reasons these techniques seem not be implemented broadly. In a Delphi consensus paper the acceptance of open preperitoneal repair was analyzed using an international survey among European Hernia Society members and a clear set of recommendations was formulated to help surgeons mastering these techniques, ensuring good patient outcomes in a practical and cost-effective manner Lorenz et al.

Like all surgical techniques, the open preperitoneal approach may not be suitable for every patient. Patient factors such as the size of the hernia, the presence of comorbidities, and the level of surgical expertise in the operating room must be carefully considered. One of these challenges is the repair of scrotal hernias. Two papers in this special issue focus on this type of more complicated inguinal hernia. Gillion et al. compared the different approaches for scrotal hernias and reflects on both the TIPP, the Lichtenstein and the laparoscopic techniques analyzing a large cohort of patients from the Club-Hernie registry, while Soler and Gillion reflects on the Minimal Open Pre-Peritoneal (MOPP) technique. Both analyses show highly acceptable outcomes for the open preperitoneal techniques, even in these more complex indication.

## Looking Forward: a Promising Future

The open preperitoneal technique represents an exciting frontier in inguinal hernia surgery. With ongoing advancements in surgical instrumentation and a better understanding of abdominal anatomy, the technique may become a more widely adopted approach, particularly as long-term data solidify its advantages. Although approach as well as outcomes are more similar to those laparoscopic approaches, the open preperitoneal techniques are often categorized with Lichtenstein and tissue-based repairs in the broad category of “open” inguinal hernia repair. In a very interesting paper by Blake et al. from the US, it is stated that these vastly different approaches together makes data Special Issue and interpretation very difficult, leaving the surgical community unable to make clinically meaningful changes to improve patient outcomes. They come up with a proposal for a new classification of inguinal hernia repair techniques, so to identify clear benefits and disadvantages, and to facilitate patients selection for a specific approach or technique.

As we move toward more patient-centered care, the open preperitoneal approach offers a promising solution for many patients suffering from inguinal hernias, combining the benefits of reduced complications, better outcomes, and fewer long-term risks. Refinement and innovation are key to improving patient care and quality of life. As with any emerging technique, it requires proper patient selection, skillful execution, and ongoing research to solidify its place as a gold standard in the field of hernia surgery.

